# Low CD86 expression is a predictive biomarker for clinical response to the therapeutic human papillomavirus vaccine IGMKK16E7: results of a post hoc analysis

**DOI:** 10.1093/jncics/pkae091

**Published:** 2024-09-20

**Authors:** Hanano Ando, Yuki Katoh, Osamu Kobayashi, Yuji Ikeda, Hideaki Yahata, Takashi Iwata, Toyomi Satoh, Azusa Akiyama, Daichi Maeda, Yumiko Hori-Hirose, Yukari Uemura, Kaori Nakayama-Hosoya, Kanoko Katoh, Takahiro Nakajima, Ayumi Taguchi, Atsushi Komatsu, Saki Kamata, Naoko Tomita, Kiyoko Kato, Daisuke Aoki, Shizunobu Igimi, Ai Kawana-Tachikawa, Danny J Schust, Kei Kawana

**Affiliations:** Department of Obstetrics and Gynecology, Nihon University School of Medicine, Tokyo, Japan; Department of Functional Morphology, Nihon University School of Medicine, Tokyo, Japan; Department of Obstetrics and Gynecology, Nihon University School of Medicine, Tokyo, Japan; Department of Obstetrics and Gynecology, Nihon University School of Medicine, Tokyo, Japan; Department of Obstetrics and Gynecology, Graduate School of Medical Sciences, Kyushu University, Fukuoka, Japan; Department of Obstetrics and Gynecology, Keio University School of Medicine, Tokyo, Japan; Department of Obstetrics and Gynecology, Faculty of Medicine, University of Tsukuba, Ibaraki, Japan; Department of Obstetrics and Gynecology, Faculty of Medicine, University of Tsukuba, Ibaraki, Japan; Department of Molecular and Cellular Pathology, Graduate School of Medical Sciences, Kanazawa University, Ishikawa, Japan; Department of Central Laboratory and Surgical Pathology, National Hospital Organization Osaka National Hospital, Osaka, Japan; Department of Data Science, Center for Clinical Science, National Center for Global Health and Medicine, Tokyo, Japan; AIDS Research Center, National Institute of Infectious Diseases, Tokyo, Japan; Department of Obstetrics and Gynecology, Nihon University School of Medicine, Tokyo, Japan; Department of Obstetrics and Gynecology, Nihon University School of Medicine, Tokyo, Japan; World Premier International Immunology Frontier Research Center, Laboratory of Human Single Cell Immunology, Osaka, Japan; Department of Obstetrics and Gynecology, Nihon University School of Medicine, Tokyo, Japan; Department of Obstetrics and Gynecology, Nihon University School of Medicine, Tokyo, Japan; Department of Obstetrics and Gynecology, Nihon University School of Medicine, Tokyo, Japan; Department of Obstetrics and Gynecology, Graduate School of Medical Sciences, Kyushu University, Fukuoka, Japan; Department of Obstetrics and Gynecology, Keio University School of Medicine, Tokyo, Japan; Department of Applied Biology and Chemistry, Tokyo University of Agriculture, Tokyo, Japan; AIDS Research Center, National Institute of Infectious Diseases, Tokyo, Japan; Department of Obstetrics and Gynecology, Duke University, Durham, NC, USA; Department of Obstetrics and Gynecology, Nihon University School of Medicine, Tokyo, Japan

## Abstract

**Background:**

Although therapeutic human papillomavirus vaccines could offer a noninvasive treatment for patients with cervical intraepithelial neoplasia, none has been clinically implemented. Oral administration of the therapeutic human papillomavirus vaccine IGMKK16E7 results in the histological regression of human papillomavirus 16–positive cervical intraepithelial neoplasia 2/3 to normal (complete response). We investigated biomarkers that could predict complete response after oral administration of IGMKK16E7.

**Methods:**

Forty-two patients administered high-dose oral IGMKK16E7 in a phase I/II trial were included. Cervix-exfoliated cells were collected before vaccine administration. Gene expression of CD4, CD8, FOXP3, programmed cell death 1 protein, CTLA4, CD103, CD28, CD80, CD86, and programmed cell death 1 ligand 1 in the cells was measured by quantitative reverse transcriptase–polymerase chain reaction. Receiver operating characteristic curve analysis and Mann-Whitney tests were used to explore potential biomarkers. Pearson correlation coefficient analysis was used to correlate gene expression profiles with clinical outcome.

**Results:**

The only predictive biomarker of vaccine response for which receiver operating characteristic curve analysis showed significant diagnostic performance with histological complete response was CD86 (area under the curve = 0.71, 95% confidence interval = 0.53 to 0.88, *P* = .020). Patients with complete response had significantly lower CD86 expression (CD86-low) than patients with no complete response (*P* = .035). The complete response rates for CD86-low and CD86-high patients were 50% and 19%, respectively, and CD86-low patients had a significantly higher complete response rate (*P* = .047). Compared with all patients, the CD86-low group had a 1.5-fold increase in the complete response rate. Gene expression of CD86 and CTLA4 showed the strongest positive correlation with clinical outcomes in the incomplete response group (*P* < .001).

**Conclusion:**

Low expression of CD86 in exfoliated cervical cells can be used as a pretreatment biomarker to predict histological complete response after IGMKK16E7 administration.

It has been demonstrated that prophylactic human papillomavirus (HPV) vaccines have dramatically reduced the risk of developing cervical cancer (1,2). Risk reductions have been demonstrated in areas where HPV vaccine coverage exceeds 50%. Therefore, to exert significant effects on the global prevalence of cervical cancer with a prophylactic HPV vaccine requires a high level of vaccine coverage in all regions of the world (3). The World Health Organization aims to eliminate cervical cancer through HPV vaccination as well as widespread cancer screening and treatment, but this outcome will take many years to achieve (4). To this point, the vast majority of cervical cancer deaths are concentrated in low- and middle-income countries (LMIC), and the mortality rate is about 10 times higher in LMIC than in more developed countries (5). Unfortunately, reaching a goal of high-level vaccine coverage is particularly problematic in LMIC, which typically do not have the economic strength to purchase vaccines or the medical resources and infrastructure to distribute them, particularly when vaccine delivery requires injection. In light of this current situation, the World Health Organization has suggested, after expert consultation, that therapeutic HPV vaccine use may help address challenges in current cervical cancer prevention programs and that the potential value of such vaccines will depend on their degree of efficacy and how quickly they can be developed and implemented relative to ongoing scale-up of existing interventions (6). Most of the therapeutic HPV vaccine candidates developed to date have reached only phase I or phase II trials; a precious few of these agents have undergone randomized, placebo-controlled trials (7-9). VGX-3100, which has been the most advanced agent in its development to date, has completed 2 phase III trials (Evaluation of VGX-3100 and Electroporation for the Treatment of Cervical HSIL [ClinicalTrials.gov identifier NCT03185013] and Evaluation of VGX-3100 and Electroporation for the Treatment of Cervical HSIL [REVEAL 2—ClinicalTrials.gov identifier NCT03721978]) (10). More mechanistic studies on VGX-3100 have reported that cervical mucosal CD137-positive, perforin-positive T cells were identified as a postexposure biomarker of VGX-3100 efficacy; no pretreatment, predictive biomarkers of therapeutic response have been reported for VGX-3100 (11). The primary endpoint of the REVEAL 2 trial using VGX-3100 was lesion regression (to cervical intraepithelial neoplasia [CIN] 1 or normal) in the posttreatment biomarker-selected population; the rate of regression to CIN-1 or normal was not significantly different from placebo. Although the secondary endpoint, regression rate for all patients, was significantly higher in the vaccinated vs placebo groups (10), the rate of regression to CIN-1 or normal in the REVEAL 2 trial was about half that of the regression rate in the phase IIb trial (12).

We conducted a phase I/II, placebo-controlled, double-blind randomized clinical trial (The mucosal immunotherapy using HPV type 16 E7–expressing Lactobacillus-based vaccine for the treatment of cervical high-grade squamous intraepithelial lesion study [MILACLE study]) in 165 patients with HPV-16–positive CIN-2/3 to determine the efficacy and safety of the therapeutic HPV vaccine IGMKK16E7 (13). IGMKK16E7 is an attenuated *Lacticaseibacillus paracasei* bacterium that displays on the bacterial cell surface a genetic fusion protein composed of mutated, full-length HPV-16 E7 and an anchoring protein derived from the lactic acid bacteria (with optimized efficiency) (14). Heat-attenuated IGMKK16E7 powder was orally administered, encapsulated in a seamless capsule. Low-dose (0.5 g/d), intermediate-dose (1.0 g/d), or high-dose (1.5 g/d) IGMKK16E7 or placebo was administered orally after fasting once each morning for 5 days during each treatment week. All patients received 4 rounds of oral immunization at weeks 1, 2, 4, and 8. In the MILACLE study, the high-dose group showed a significantly higher complete response (ie, histological regression to normal) rate than the placebo group (high-dose vs placebo: 31.7% vs 12.5%, 95% confidence interval [CI] = 0.5 to 37.8). In patients with CIN-2/3 positive for HPV-16 alone, the complete response rate was 40.0% in the high-dose group and 11.5% in the placebo group (95% CI = 4.3 to 50.0). Furthermore, the number of HPV-16 E7–specific interferon gamma (IFN-γ)–producing cells (spot-forming cells) among peripheral blood mononuclear cells increased with treatment response. Complete response rates were dose dependent among hyper-responders to IGMKK16E7 (13).

IGMKK16E7 is an orally administered vaccine that fulfills an unmet need for noninvasive therapy for CIN-2/3, making it a favorable vaccination strategy for LMIC with limited medical resources. For oral vaccines, it is difficult to examine biomarkers of response in the priming phase because the priming phase occurs in the intestinal mucosa. In contrast, clinical specimens can easily be collected from the cervical mucosa, including CIN-2/3 lesions, allowing measurement of biomarkers in the effector phase.

In this study, we investigated the pretreatment local immune environment at the cervical mucosa in patients who received high doses of IGMKK16E7 that exhibited clinical efficacy. This study was designed post hoc and in response to the results of the MILACLE study. To examine the correlation between IGMKK16E7 administration and clinical efficacy in the effector phase, the 42 patients who took high vaccine doses and demonstrated clinical efficacy were included in this study. By comparing the immunological characteristics of cervical cells between individuals with and without complete response, we explored possible predictive biomarkers that could allow gut-derived, E7-specific T cells to operate effectively in the effector phase. The aim of this study was to determine which cervical immune biomarkers could predict histological regression of cervical dysplasia after IGMKK16E7 use.

## Methods

### The MILACLE study

The mucosal immunotherapy using a HPV-16 E7–expressing, *Lactobacillus*-based vaccine for the treatment of cervical high-grade squamous intraepithelial lesion study (MILACLE study) was a randomized, double-blind, placebo-controlled clinical trial conducted at 4 centers in Japan (Japan Registry Clinical Trial identifier jRCT2031190034) (13). The clinical trial was supported by grants from the Japan Agency for Medical Research and Development (AMED) and GLVOVACC Co Ltd; the funding agencies had no influence on study design or trial implementation, and were not involved in data collection or analysis, in the writing of the manuscript, or in the decision to submit it for publication. The trial was performed in accordance with the principles of the Declaration of Helsinki.

### Patients

Patients with pathologically determined CIN-2 or 3 who were positive for HPV-16 by HPV genotyping using exfoliated cervical cells were eligible for enrollment. Patients aged 20 to 45 years were included (13,15). Participants were randomly assigned in a 1:1:1:1 ratio to the low-dose, intermediate-dose, or high-dose IGMKK16E7 groups or to the placebo group. The target number of participants in this clinical trial was set at 41 persons for each group. Pathological evaluation (colposcopy-directed biopsy) was performed 16 or 24 weeks after the first dose. The final enrollment of the MILACLE study was 165 participants, of whom 43 were assigned to the high-dose group (13). Of these 43 individuals, 42 were eligible for this study because pathological evaluation and measurement of cervical cells were available. Patients who regressed to normal, patients who regressed to CIN-1, and patients who remained at CIN-2/3 by histological evaluation at 16 or 24 weeks were classified as having complete response, partial response, or stable disease, respectively. In this trial, complete response was defined as histological regression to normal, but viral clearance was not always achieved in the complete response patients. In fact, viral clearance could not be demonstrated in the parent MILACLE study based on its predetermined study size and chosen clinical endpoints.

### Clinical samples and biological assays

Exfoliated cervical cells for immunological evaluation were sampled in outpatient clinics before registration and at 16 and 24 weeks after the first study dose. Exfoliated cells were collected by abrading the cervix with a spatula; specimens were then suspended in a preservative solution of 35% to 55% methanol. Exfoliated cervical cells collected before registration were used in this study. Because IGMKK16E7 is an orally administered vaccine, the priming phase is considered to occur in the intestinal mucosal lymphoid tissue; however, because it is difficult to collect lymphocytes from the intestine, peripheral blood mononuclear cells were substituted and evaluated in the parent MILACLE study (13).

Quantitative reverse transcriptase–polymerase chain reaction (RT-PCR) using exfoliated cervical cells was performed according to standard protocols. Total RNA was isolated from exfoliated cervical cells using the miRNeasy Mini Kit (QIAGEN, Hamburg, Germany). Immune-related factors, including CD4, CD8, CD28, CD80, CD86, CD103, FOXP3, programmed cell death 1 protein (PD-1), programmed cell death 1 ligand 1 (PD-L1), and CTLA4, were evaluated by the 2^-ΔCt^ method. In brief, the relative quantification value is expressed as 2^−ΔCt^, in which ΔCt is the difference between the mean Ct value of duplicate measurements of the sample and the endogenous β-actin control. TaqMan quantitative PCR primers and probes were purchased from Applied Biosystems. With the exception of PD-1, gene expression measurements were performed using purchased primer sets (Supplementary Table 1, available online); primers from previous studies were used for PD-1 (16).

### Statistical analysis

Biomarkers predicting histological regression were analyzed using receiver operating characteristic (ROC) curves. Sensitivity, specificity, and area under the curve were obtained from ROC curves. Cutoff values were determined using the closest-to-(0,1) criterion (17). Complete response and incomplete response groups for each biomarker were compared for statistical significance by Mann-Whitney *U* testing. Outcomes for histological regression were analyzed by Fisher exact testing. Correlations among the molecules were analyzed using the Pearson product-moment correlation coefficient (Pearson *r*). All analyses were performed using full-analysis sets. Two-sided *P*  less than .05 was considered statistically significant. Analyses were performed in Excel 2015 software (Microsoft Corp, Redmond, WA); SAS, version 9.4, software (SAS Institute Inc, Cary, NC); and R, version 3.3.1, software (R Foundation for Statistical Computing, Vienna, Austria).

## Results

### Patient characteristics

Among 42 patients in whom high-dose IGMKKE7 was orally administered, 11 and 31 patients were diagnosed as having CIN-2 and CIN-3, respectively; 31% (13/42) of patients with CIN-2/3 were positive for HPV-16 only, and the remaining 29 patients (69%) were positive for HPV-16 and other HPV genotypes. In the MILACLE study, histological complete response occurred in 13 of 42 (31%) high-dose recipients; the remaining 29 patients (69%) were diagnosed histologically as not having a complete response (partial response + stable disease).

### Pretreatment biomarkers of histological complete response

**Figure 1.**
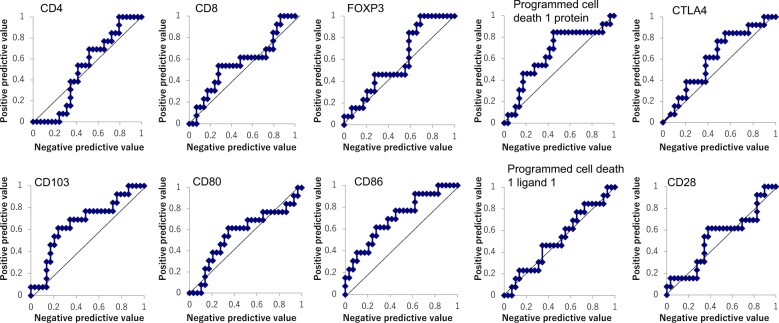
Receiver operating characteristic curves for each biomarker considered for prediction of histological complete response (regression to normal): quantitative reverse transcriptase–polymerase chain reaction values for each biomarker from cervical cells obtained from each patient exposed to high-dose IGMKK16E7. Positive predictive values and negative predictive values were plotted for diagnosis of histological complete response. The point farthest from the straight line was used as the cutoff value.

The association between histological complete response and gene expression of biomarkers in the cervical mucosa, the site of the vaccine effector phase, was analyzed by diagnostic ROC curves (Table 1, Figure 1). Gene expression of each biomarker was measured by quantitative RT-PCR using exfoliated cervical cells collected from patients before oral administration of IGMKK16E7. CD4, CD8, FOXP3, PD-1, CTLA4, CD103 (integrin αEβ7), and CD28 were measured as lymphocyte-related factors, and CD80, CD86, CD103, and PD-L1 were measured as antigen-presenting cell–related or epithelial cell–related factors. CD103 is expressed on intraepithelial lymphocytes, submucosal lymphocytes, Tregs, and dendritic cells.

**Table 1.** Receiver operating characteristic curve analysis for biomarkers to predict histological complete response after IGMKK16E7 administration

Factor	Area under the curve (95% CI)	SD	Odds ratio	*P*
CD4	0.51 (0.33 to 0.68)	0.090	2.1	.94
CD8	0.56 (0.36 to 0.76)	0.10	3.1	.56
FOXP3	0.60 (0.42 to 0.78)	0.092	2.3	.29
Programmed cell death 1 protein	0.65 (0.46 to 0.84)	0.097	6.8	.13
CTLA4	0.62 (0.43 to 0.80)	0.093	3.6	.21
CD103	0.66 (0.48 to 0.85)	0.094	5.0	.084
CD28	0.53 (0.33 to 0.73)	0.10	2.6	.75
CD80	0.57 (0.37 to 0.77)	0.10	3.0	.51
**CD86** ^*^	**0.71 (0.53 to 0.88)**	**0.089**	**4.2**	**.020**
Programmed cell death 1 ligand 1	0.52 (0.33 to 0.71)	0.098	1.6	.82

* Asterisk indicates a significant correlation.

CD86 levels were most strongly associated with histological complete response. This association was significant (area under the curve = 0.71, 95% CI = 0.53 to 0.88, *P* = .020). CD86 performed as a predictive biomarker of complete response, with 62% sensitivity and 72% specificity. Cervical CD103 levels were also associated with histological complete response but with marginal statistical significance (area under the curve = 0.66, 95% CI = 0.48 to 0.85, *P* = .084). No other biomarkers were found to correlate in a way that could predict histological complete response.

**Figure 2.**
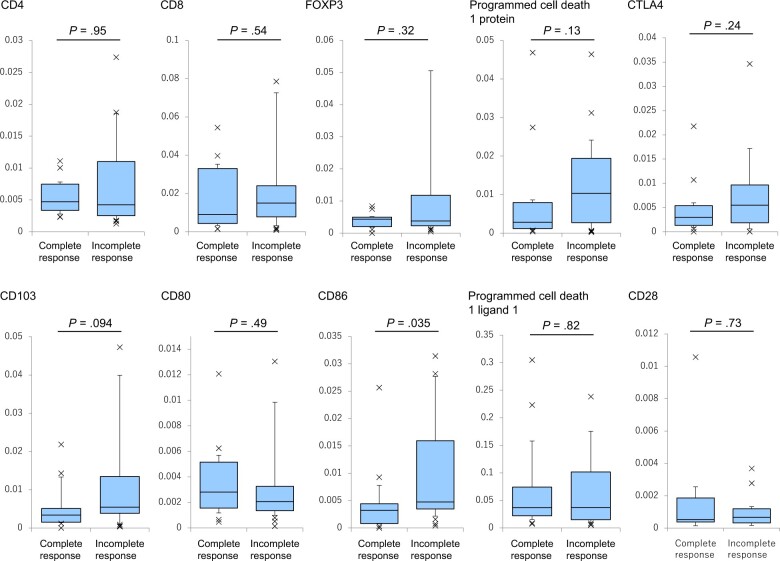
Reverse transcriptase–polymerase chain reaction (RT-PCR) values of gene expression levels for each biomarker for the complete response and incomplete response groups: 5-number summary using box and whisker plot. The horizontal bar is the median, with boxes from the first quartile (25th percentile) to the third quartile (75th percentile) across the median and a whisker at 1.5 times the IQR. Cross-marks indicate RT-PCR values that deviated from the box. Mann-Whitney *U* testing was performed to compare RT-PCR values for each biomarker in the 2 groups (*P* values in Figure 2).

In Figure 2, RT-PCR gene expression levels of each biomarker for each complete response and incomplete response group are depicted and compared in 5-number summaries using box and whisker plots. Mann-Whitney *U* testing was performed to compare RT-PCR values for each biomarker for 2 groups: complete response and incomplete response (Figure 2). Only CD86 levels differed significantly between the 2 groups, with patients experiencing complete response having significantly lower CD86 gene expression levels than patients without complete response (*P* = .035). CD103 levels also showed a nonsignificant trend toward lower gene expression in the complete response group compared with the incomplete response group (*P* = .094). Gene expression levels for each patient in the 2 groups are shown in dot plots (Supplementary Figure 1, available online).

### Histological complete response rates in patients with low cervical CD86 expression (CD86-low)

**Figure 3.**
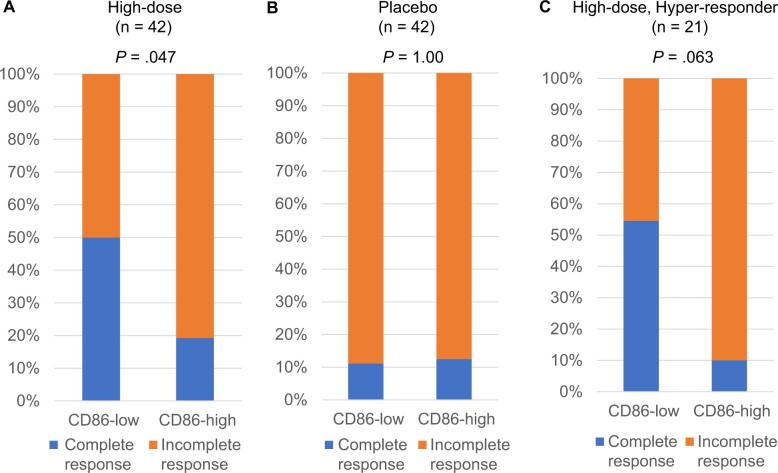
Histological complete response (regression to normal) rates in patients with CD86-low vs CD86-high status: A CD86 cutoff value was established from receiver operating characteristic curve analysis, and higher values were defined as CD86-high and lower values as CD86-low. The complete response rates for each group are shown in bar graphs for the total population in the high-dose group (n = 42), the total population in the placebo group (n = 42), and the hyper-responder group (n = 21). Comparison of complete response rates between the 2 groups was done by Fisher exact test (*P* value).

Cutoff values for CD86 obtained from the ROC curves in Figure 1 were used to subclassify patients with low CD86 gene expression (CD86-low) and high CD86 gene expression (CD86-high). In the high-vaccine-dose group, CD86-low patients had a significantly higher complete response rate (*P* = .047), with a complete response rate of 50% for CD86-low vs 19% for CD86-high patients. The complete response rate for patients showing low levels of CD86 was 1.5-fold higher than the 31% rate for all high-dose patients (Figure 3, A). We also examined whether the association between cervical CD86-low status, as defined by our ROC curves, and histological complete response was similar in patients in the placebo group (Figure 3, B). Because the complete response rate was unchanged (13% vs 14% in CD86-low vs CD86-high groups, respectively) in relation to CD86 gene expression levels in the placebo group, the tendency of CD86-low patients to have a complete response was specific to oral administration with IGMKK16E7. The rates of spontaneous regression to normal from CIN-2/3 noted in placebo-controlled trials are generally around 10% (7,8,12,13). To investigate whether CD86-low patients with no histological complete response had lower levels of E7-specific T cells during the priming phase, we examined the complete response rate of CD86-low patients in hyper-responders (Figure 3, C). Here, *hyper-responder* refers to patients with a spot-forming cell change that was higher than the median value for all patients; the complete response rate of hyper-responders increased in a dose-dependent manner. The complete response rate in the 21 identified hyper-responders was 55% for CD86-low and 10% for CD86-high (*P* = .064) individuals. Despite high levels of E7-specific circulating T cells induced by IGMKK16E7, 90% of CD86-high patients did not have histological complete response.

### Characteristics of the cervical cells isolated from CD86-low and CD86-high patients

**Figure 4.**
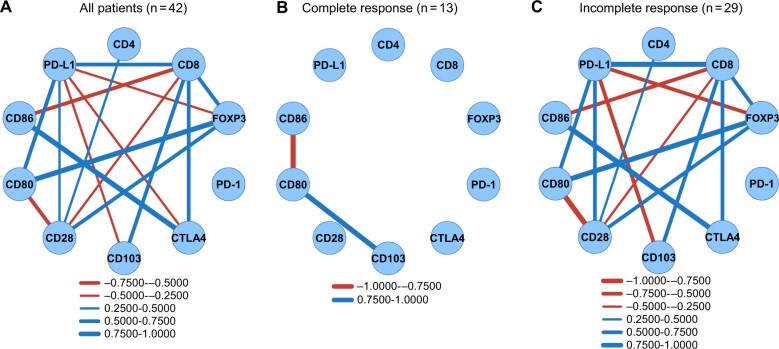
Multifactorial relevance of cervical gene expression levels for proposed biomarkers: Significant correlations in the expression of each immune molecule were connected by solid lines, the thickness of which indicated the strength of the correlation. **A)** All eligible patients (n = 42), **(B)** patients with histological complete response (n = 13), and **(C)** patients with histological incomplete response (n = 29). Solid bold numbers indicated Pearson *r* values. The correlation was analyzed by Pearson product-moment correlation coefficient. PD-1 = programmed cell death 1 protein; PD-L1 = programmed cell death 1 ligand 1.

To investigate the reason why patients with cervical cells with high CD86 expression did not exhibit histological complete response, the multifactorial relevance of gene expression patterns at the cervix was analyzed by Pearson product-moment correlation coefficients. The gene expression levels for each of the assayed molecules that showed positive or negative correlations are depicted in Supplementary Figure 2 (available online). Significantly correlated gene expression levels are connected by solid bonds, the thickness of which indicates the strength of the correlation (Figure 4). Among these, CD86 and CTLA4 showed the strongest positive correlation (*P* < .001, *r* = 0.77). CD86 had a negative correlation with CD8 (*P* < .001, *r* = ‒0.53) but no correlation with CD28. CD8 correlated positively with CTLA4 (*P* < .001, *r* = 0.56), CD103 (*P* < .001, *r* = 0.67), and PD-L1 (*P* < .001, *r* = 0.74). Correlations between the gene expression levels of several immune molecules was further examined in the complete response and incomplete response groups (Figure 4, B and 4, C). In the complete response group, CD86, CD80, and CD103 showed a strong correlation, even though the small number of patients made it difficult to find significant differences. A negative correlation (*P* = .019, *r* = ‒0.94) was shown between CD86 and CD80, while CD80 showed a positive correlation with CD103 (*P* = .034, *r* = 0.91). CD86 did not correlate with CTLA4 in the cervices of complete response patients. In contrast, there were strong positive correlations between CD86 and CTLA4 (*P* < .001, *r* = 0.83), CD8 and PD-L1 (*P* < .001, *r* = 0.81), and CD80 and FOXP3 (*P* < .001, *r* = 0.81) and a negative correlation between CD80 and CD28 (*P* < .001, *r* = ‒0.79) in the cervices of patients with no complete response.

**Figure 5.**
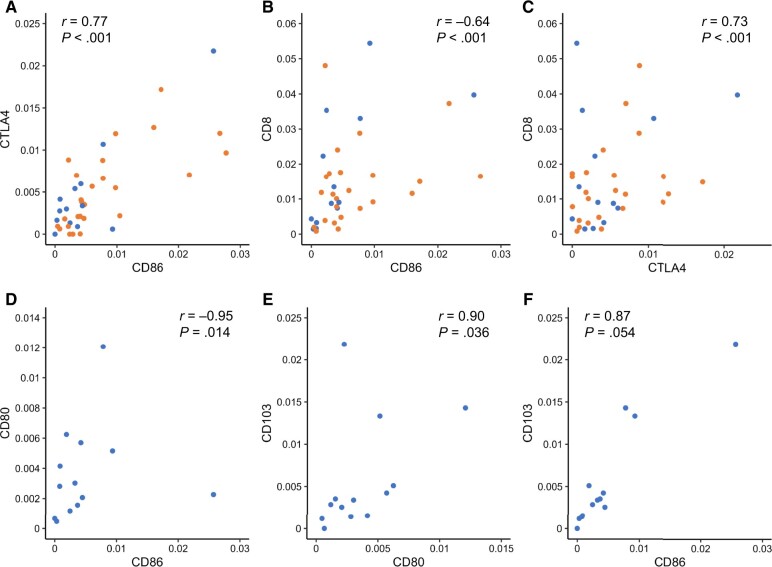
Scatterplot depictions of reverse transcriptase–polymerase chain reaction values by patient to identify correlations between sets of biomarkers (upper panel, all patients; lower panel, patients with histological complete response): **(A)** correlation between CD86 and CTLA4, **(B)** correlation between CD86 and CD8, **(C)** correlation between CTLA4 and CD8, **(D)** correlation between CD86 and CD80, **(E)** correlation between CD80 and CD103, and **(F)** correlation between CD86 and CD103. Orange and blue plots were incomplete response and complete response patients, respectively. The *r* value is the correlation coefficient (Pearson *r*), and *P* is the *P* value by Pearson product-moment correlation coefficient.

These correlations were further confirmed using scatter plots (Figure 5: top panels, all patients; bottom panels, complete response patients). Figure 5, A shows that CD86 and CTLA4 are strongly and positively correlated when all patients are included. In contrast, CD86 and CD8 showed a negative correlation (Figure 5, B), and CTLA4 and CD8 showed a positive correlation (Figure 5, C). In the lower panels of Figure 5, the RT-PCR values of CD80, CD86, and CD103 were lower in complete response patients than those for all patients combined, and CD86, CD80, and CD103 were positively correlated among these low values (Figure 5, D-5, F).

## Discussion

We found that low cervical CD86 levels before treatment are a predictive biomarker of response to the therapeutic oral HPV vaccine IGMKK16E7 in HPV16-positive CIN-2/3. Individuals with CD86-high status in the local cervical microenvironment were unlikely to display histological complete response. In patients selected for CD86-low status, the complete response rate with IGMKK16E7 increased to 50% and may therefore be a useful diagnostic biomarker for selecting patients with the highest likelihood of realizing optimal clinical efficacy after IGMKK16E7 use. Here, we report on a post hoc analysis of the IGMKK16E7 MILACLE study, with particular focus on those patients taking high oral vaccine doses who demonstrated clinical efficacy, to most efficiently identify candidate predictive biomarkers of vaccine response. Therefore, the number of patients in this study is limited to 42, and evaluation of CD86-low status as a predictive biomarker requires a follow-up validation study, with a planned number of patients determined using the Reporting Recommendations for Tumor Marker Prognostic Studies guidelines.

In this study, specimens from cytology or HPV genotyping tests, which are frequently used in routine practice, were diverted for clinical testing for predictive biomarkers of IGMKK16E7 response. The components of cervical specimens include cervical epithelial cells containing CIN-2/3 neoplastic cells, leukocytes, and antigen-presenting cells. Because the ratios of these components in cervical specimens fluctuate across the menstrual cycle, measuring specific gene expression levels by cell subtype is difficult. Instead, by comprehensively sampling exfoliated cervical cells, we measured biomarkers in the total population of cells residing in the cervical mucosa, including those in CIN lesions.

The B7 family (CD80, CD86) of molecules are co-stimulatory ligands exposed on the cell surface of antigen-presenting cells, including macrophages, dendritic cells, Langerhans cells, B cells, and epithelial cells. CD80 and CD86 act in T-cell activation by binding to CD28 and in T-cell suppression by binding to CTLA4, but CD80 and CD86 are expressed in different cell types and are often differentially regulated in them (18). CD86 gene expression is induced in dendritic cells and Langerhans cells by interferon gamma as well as bacterial and viral infections (19). Most antigen-presenting cells constitutively express low levels of CD86 and rapidly upregulate CD86 expression after activation. In contrast, CD80 expression is not induced immediately but is delayed (18). In this study, we did not detect a positive correlation between CD80 and CD86 expression in the total patient population or in any of the subgroups in this study. Monomeric CD86 binds to CTLA4 with low affinity, and this binding not only competes with CD28 for binding to CD80 and CD86 but also inhibits T-cell activation by removing CD80 and CD86 itself from the cell surface of antigen-presenting cells by transendocytosis (20). When the T-cell receptor is strongly stimulated by antigens, CTLA4 expression is upregulated by increased transport to the cell surface from intracellular stores (21).

In our dataset, gene expression levels for CD86 and CTLA4 were strongly positively correlated. Although gene expression of CD86 and CTLA4 is not directly linked, our results suggested that in local environments where these 2 genes are highly expressed, T-cell activation is inhibited in the effector phase, resulting in no complete response or immunological regression. Because T-cell suppression by CTLA4 is antigen independent, an environment with high CD86/CTLA4 gene expression, as seen in some of our patients, was not related to HPV antigen but was instead characteristic of the cervices of patients with incomplete response to vaccination. Cervical lymphocytes and cervical epithelial cells cannot be used for flow cytometry and cannot self-renew, making their use for in vitro experiments difficult. Interpretation of the CD86/CTLA4 gene expression correlation is still hypothetical because the cell populations expressing CD86 and CTLA4 have not been definitively identified. In the IGMKK16E7 clinical trial, paraffin sections of tissue samples were not collected from patients, so detailed immunostaining or other pathological analysis of tissue samples could not be performed. This is a limitation of this study.

Studies have examined the effect of CD86 on CIN and cervical cancer: CD80/CD86-positive dendritic cells are reported to be more abundant in the cervices of women with high-risk HPV positivity but no CIN lesions (22). In addition, immunostaining of normal cervical epithelial cells has shown that CD86 is positive and expressed in the superficial and intermediate layers rather than the basal layer of the stratified squamous epithelium (23). It is known that CD86 but not CD80 is expressed in normal epithelial cells and from cells obtained from patients with CIN (24). In CIN-1 lesions, CD86 expression is reported to be downregulated compared with normal epithelium, and patients who remain CIN-1 positive may be more susceptible to immune induction in a CD86-low environment. The specimens used in this study were part of liquid cytology specimens. Because such specimens generally contain many more epithelial cells than antigen-presenting cells, most of the cellular components in our specimens were likely cervical epithelial cells. Taken together, we hypothesize that low gene expression of CD86 in liquid cytology samples may reflect expression in the cervical epithelial cells rather than in antigen-presenting cells. Immunostaining for CD86 and CD80 in tissue and liquid cytology specimens of a typical CIN lesion is shown in Supplementary Figure 3 (available online), and it can be seen that CD86 is present on epithelial cells.

It is a limitation of this study that the cell of origin of the genes analyzed, their posttranscriptional regulation, and their cell surface expression are unknown. CD103 is expressed on intraepithelial lymphocytes, submucosal lymphocytes, Tregs, and dendritic cells and is associated with homing and local immunity. These characteristics have been documented in the priming and effector phases of the intestinal tract and skin, respectively (25). The fact that the complete response group tended to have lower CD103 expression in our results may indicate that patients with fewer CD103-associated immune cells in the cervix before treatment may mount a stronger effector-phase response when immune cells are first induced in the intestinal tract by IGMKK16E7 administration. In the complete response patients, CD103 and CD86 values were low, but even these low values were correlated, which may suggest the presence of a small number of CD103-positive dendritic cells in which CD86 and CD103 are expressed together.

Assuming that high gene expression of CD86/CTLA4 results in incomplete response because of T-cell suppression at the cervix, immune checkpoint evasion with anti-CTLA4 antibodies may enhance the efficacy of IGMKK16E7, especially for CD86-high patients. Combination therapy with cancer vaccines and anti-CTLA4 antibodies for malignant tumors has been the subject of numerous clinical trials (26). In the phase II clinical trial of the pembrolizumab plus GX-188E therapeutic DNA vaccine in patients with HPV-16–positive or HPV-18–positive advanced cervical cancer, 42% of the 26 patients receiving vaccine had clinical responses (27). IGMKK167 can be considered a cancer vaccine targeting the tumor antigen HPV-16 E7. Clinical trials to investigate whether the combination therapy of IGMKK16E7 and anti-CTLA4 antibody has antitumor effects against invasive cervical cancer and other HPV-related cancers will be considered in the future, as will efforts to establish additional predictive biomarkers that allow personalized therapy with IGMKK16E7.

## Trial registration

Japan Registry Clinical Trial identifier jRCT2031190034.

## Supplementary Material

pkae091_Supplementary_Data

## Data Availability

Participant data can be shared with outside collaborators for research to understand more about the clinical efficacy and safety of IGMKK16E7 and immune responses to the vaccine obtained from the MILACLE study. These data are available online at https://www.nichidaisanfujinka.com/milaclestudy.
