# Supplementary material for: Low CD86 expression is a predictive biomarker for clinical response to the therapeutic human papillomavirus vaccine IGMKK16E7: results of a post hoc analysis
Source: JNCI Cancer Spectr. 2024 Sep 20;8(6):pkae091. doi: 10.1093/jncics/pkae091 (PMC11528511; doi:10.1093/jncics/pkae091)
Supplement: pkae091_Supplementary_Data [file pkae091_supplementary_data.zip › AndoH_CD86biomarkerJNCICS_SuppleTable10623.pdf]

Supplementary table

Supplementary Table 1

Supplementary Table 1 List of primer set for PCR to detect gene expression of each biomarker

	Assay ID	Sequences for primers and probe
CD4*	Hs01058407_m1	
CD8*	Hs00233520_m1	
CD28*	Hs01007422_m1	
CD80*	Hs01045161_m1	
CD86*	Hs01567026_m1	
CD103*	Hs01025372_m1	
Foxp3*	Hs01085834_m1	
PD-L1*	Hs00204257_m1	
CTLA-4*	Hs00175480_m1	
		5'→3'
	Forward primer	AGGCATGCAGATCCCACA
PD-1**	Reverse primer	CCTGTCTGGGGAGTCTAAGA
	Probe	TCTGGGCGGTGCTACAACT

*CD4, CD8, CD28, CD80, CD86, CD103, Foxp3, PD-L1, CTLA-4 are detected using commercial-based primers.

**PD-1 is detected using above primers and probe according to Reference 16.
