# Supplementary material for: Low CD86 expression is a predictive biomarker for clinical response to the therapeutic human papillomavirus vaccine IGMKK16E7: results of a post hoc analysis
Source: JNCI Cancer Spectr. 2024 Sep 20;8(6):pkae091. doi: 10.1093/jncics/pkae091 (PMC11528511; doi:10.1093/jncics/pkae091)
Supplement: pkae091_Supplementary_Data [file pkae091_supplementary_data.zip › AndoH_MILACLE_CD86_SupplementaryFigures.pdf]

Low CD86 expression is a predictive biomarker for clinical response to the therapeutic HPV vaccine, IGMKK16E7

Hanano Ando et al.

Supplementary Figures

1. **Supplementary Figure 1**

2. **Supplementary Figure 2**

3. **Supplementary Figure 3**

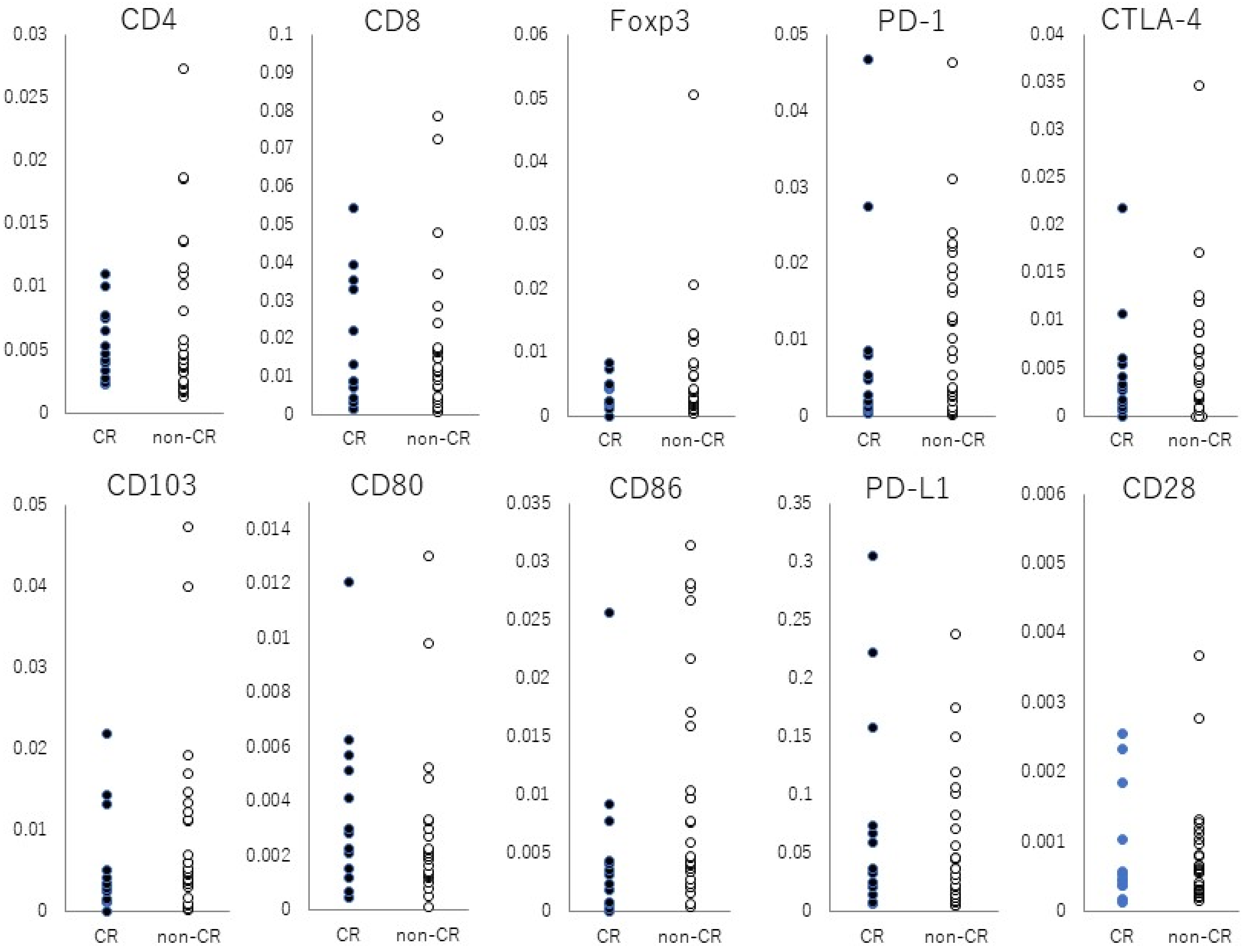

Supplementary Figure 1
RT-PCR values of gene expression levels of each biomarker for the CR and non-CR groups: Gene expression levels for each patient in the two groups are shown in dot plots.

All patients (n=42)

Pearson's product-moment correlation coefficient [*,P<0.05 **,P<0.01]

	CD4	CD8	Foxp3	PD-1	CTLA4	CD103	CD28	CD80	CD86	PD-L1
CD4	-	0.9801	0.9346	0.8718	0.0641	0.1950	0.0063	0.4693	0.9955	0.9034
CD8		-	0.0013	0.5835	P < 0.001	P < 0.001	0.0327	0.0604	0.0013	P < 0.001
Foxp3		**	-	0.3052	0.6950	0.8720	P < 0.001	P < 0.001	0.6671	0.0040
PD-1				-	0.3280	0.2449	0.3482	0.4708	0.4905	0.9320
CTLA4		**			-	0.1665	0.1035	0.1864	P < 0.001	0.0318
CD103		**				-	0.3031	0.9593	0.0907	0.0056
CD28	**	*	**				-	P < 0.001	0.3006	0.0060
CD80			**				**	-	0.2153	P < 0.001
CD86		**			**				-	0.0581
PD-L1		**	**		*	**	**	**		-

Pearson's product-moment correlation coefficient

	CD4	CD8	Foxp3	PD-1	CTLA-4	CD103	CD28	CD80	CD86	PD-L1
CD4	1.0000	0.0044	-0.0146	-0.0287	0.3211	-0.2279	0.4595	0.1284	0.0010	0.0216
CD8	0.0044	1.0000	0.5300	-0.0974	0.5675	0.6729	-0.3672	-0.3254	-0.5302	0.7413
Foxp3	-0.0146	0.5300	1.0000	-0.1812	0.0698	0.0287	0.6567	0.7502	0.0765	-0.4811
PD-1	-0.0287	-0.0974	-0.1812	1.0000	0.1730	0.2050	0.1660	0.1279	0.1224	-0.0152
CTLA4	0.3211	0.5675	0.0698	0.1730	1.0000	-0.2428	-0.2840	-0.2322	0.7698	-0.3690
CD103	-0.2279	0.6729	0.0287	0.2050	-0.2428	1.0000	0.1819	-0.0091	0.2947	-0.4649
CD28	0.4595	-0.3672	0.6567	0.1660	-0.2840	0.1819	1.0000	-0.6785	0.1829	0.4618
CD80	0.1284	-0.3254	0.7502	0.1279	-0.2322	-0.0091	-0.6785	1.0000	0.2181	0.5572
CD86	0.0010	-0.5302	0.0765	0.1224	0.7698	0.2947	0.1829	0.2181	1.0000	0.3282
PD-L1	0.0216	0.7413	-0.4811	-0.0152	-0.3690	-0.4649	0.4618	0.5572	0.3282	1.0000

CR (n=13)

Pearson's product-moment correlation coefficient [*,P<0.05 **,P<0.01]

	CD4	CD8	Foxp3	PD-1	CTLA4	CD103	CD28	CD80	CD86	PD-L1
CD4	-	0.8165	0.1993	0.1206	0.5145	0.1377	0.4085	0.1873	0.2441	0.5144
CD8		-	0.8103	0.8221	0.4065	0.5671	0.9626	0.9237	0.9090	0.2005
Foxp3			-	0.2459	0.3006	0.2332	0.1352	0.3117	0.4648	0.5756
PD-1				-	0.7750	0.1126	0.5757	0.1303	0.1269	0.5949
CTLA4					-	0.6152	0.1599	0.9921	0.7359	0.8689
CD103						-	0.4443	0.0340	0.0750	0.6954
CD28							-	0.5912	0.7869	0.5544
CD80						*		-	0.0187	0.5229
CD86								*	-	0.4358
PD-L1										-

Pearson's product-moment correlation coefficient

	CD4	CD8	Foxp3	PD-1	CTLA4	CD103	CD28	CD80	CD86	PD-L1
CD4	1.0000	0.1446	-0.6878	-0.7788	0.3916	-0.7579	0.4842	0.7009	0.6407	0.3917
CD8	0.1446	1.0000	0.1496	0.1402	-0.4860	0.3471	0.0294	-0.0600	0.0716	0.6866
Foxp3	-0.6878	0.1496	1.0000	-0.6389	0.5846	-0.6520	0.7609	0.5738	0.4345	0.3400
PD-1	-0.7788	0.1402	-0.6389	1.0000	0.1777	-0.7890	0.3399	0.7669	0.7710	0.3239
CTLA4	0.3916	-0.4860	0.5846	0.1777	1.0000	0.3071	-0.7318	-0.0062	0.2090	0.1032
CD103	-0.7579	0.3471	-0.6520	-0.7890	0.3071	1.0000	0.4524	0.9061	0.8399	0.2416
CD28	0.4842	0.0294	0.7609	0.3399	-0.7318	0.4524	1.0000	-0.3270	-0.1682	-0.3578
CD80	0.7009	-0.0600	0.5738	0.7669	-0.0062	0.9061	-0.3270	1.0000	-0.9373	-0.3844
CD86	0.6407	0.0716	0.4345	0.7710	0.2090	0.8399	-0.1682	-0.9373	1.0000	-0.4599
PD-L1	0.3917	0.6866	0.3400	0.3239	0.1032	0.2416	-0.3578	-0.3844	-0.4599	1.0000

Non-CR (n=29)

Pearson's product-moment correlation coefficient [*,P<0.05 **,P<0.01]

	CD4	CD8	Foxp3	PD-1	CTLA4	CD103	CD28	CD80	CD86	PD-L1
CD4	-	0.6306	0.6935	0.9606	0.3850	0.2516	0.0409	0.4617	0.8695	0.7249
CD8		-	0.0038	0.4720	0.0021	P < 0.001	0.0222	0.0598	0.0056	P < 0.001
Foxp3		**	-	0.3345	0.9267	0.8336	P < 0.001	P < 0.001	0.4966	0.0046
PD-1				-	0.1673	0.1750	0.3293	0.3266	0.8108	0.9383
CTLA4		**			-	0.0984	0.4556	0.3817	P < 0.001	0.0725
CD103		**				-	0.2625	0.7688	0.1565	0.0093
CD28	*	*	**				-	P < 0.001	0.6412	0.0031
CD80			**				**	-	0.4445	0.0016
CD86		**			**				-	0.1219
PD-L1		**	**			**	**	**		-

Pearson's product-moment correlation coefficient

	CD4	CD8	Foxp3	PD-1	CTLA4	CD103	CD28	CD80	CD86	PD-L1
CD4	1.0000	0.1114	-0.0914	0.0115	0.1999	-0.2618	0.4495	0.1699	0.0382	-0.0817
CD8	0.1114	1.0000	0.6029	-0.1660	0.6329	0.7031	-0.4959	-0.4174	-0.5825	0.8104
Foxp3	-0.0914	0.6029	1.0000	-0.2215	-0.0214	-0.0488	0.7448	0.8109	0.1571	-0.5931
PD-1	0.0115	-0.1660	-0.2215	1.0000	0.3129	0.3076	0.2239	0.2251	-0.0556	0.0180
CTLA4	0.1999	0.6329	-0.0214	0.3129	1.0000	-0.3703	-0.1722	-0.2013	0.8334	-0.3998
CD103	-0.2618	0.7031	-0.0488	0.3076	-0.3703	1.0000	0.2561	0.0682	0.3206	-0.5529
CD28	0.4495	-0.4959	0.7448	0.2239	-0.1722	0.2561	1.0000	-0.7923	0.1080	0.6131
CD80	0.1699	-0.4174	0.8109	0.2251	-0.2013	0.0682	-0.7923	1.0000	0.1763	0.6449
CD86	0.0382	-0.5825	0.1571	-0.0556	0.8334	0.3206	0.1080	0.1763	1.0000	0.3482
PD-L1	-0.0817	0.8104	-0.5931	0.0180	-0.3998	-0.5529	0.6131	0.6449	0.3482	1.0000

Heat map of partial correlation coefficient

1.00	-1.00～-0.75
0.75～1.00	-0.75～-0.50
0.50～0.75	-0.50～-0.25
0.25～0.50	-0.25～0.00
0.00～0.25	

Supplementary Figure 2

The gene expression levels for each of the assayed molecules that showed positive (blue) or negative (red) correlations are depicted in right panel. P values are depicted in left panel. One and two asterisks indicates p<0.05 and p<0.01, respectively.

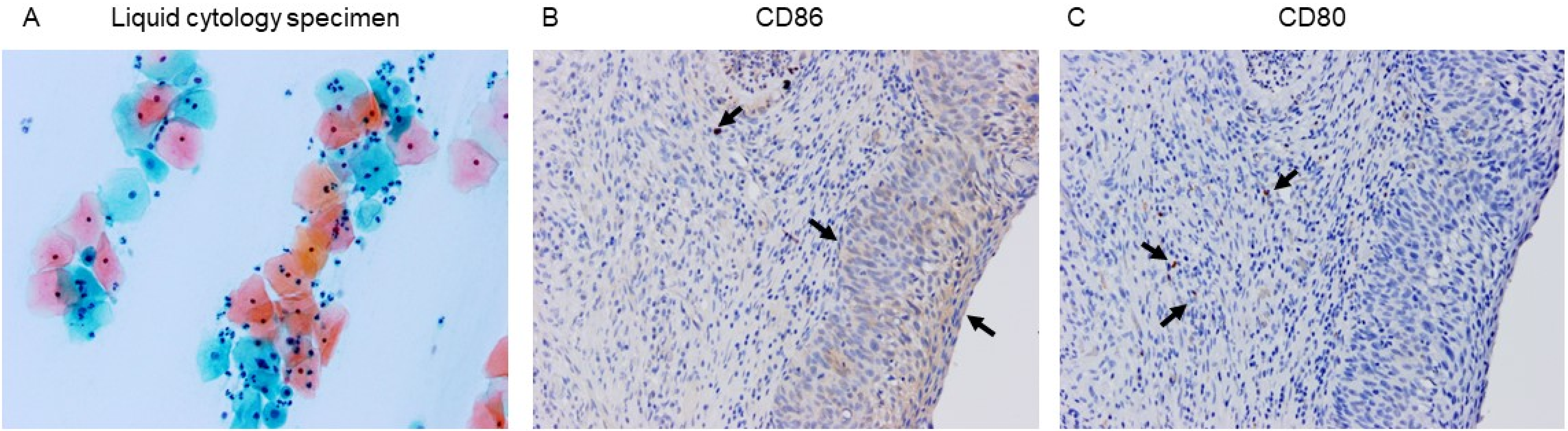

Supplementary Figure 3

Pictures of liquid cytology specimens (Papanicolaou stain, 400x) and immunostaining for CD86 and CD80 (100x) of a representative CIN3 case. As indicated in A, the liquid cytology specimens we used for biomarker study are predominantly epithelial cells, with some mononuclear cells and neutrophils. B: CD86 was expressed in epithelial cells. CD86-positive cells were observed in the submucosal tissue. C: CD80 was almost never expressed in epithelial cells although CD80-positive cells were observed in the submucosal tissue.
